# Spatial patterns and risk mapping of opisthorchiasis and soil-transmitted helminth infections in Thailand using Bayesian geostatistical models

**DOI:** 10.1038/s41598-026-55420-0

**Published:** 2026-06-11

**Authors:** Khanittha Pratumchart, Apiporn T. Suwannatrai, Oranard Wattanawong, Kavin Thinkhamrop, Kulwadee Suwannatrai, Ampas Wisetmora, Suphansa Wichairattanagull, Nichaphat Kukitirat, Thitima Wongsaroj, Pariwate Varnakovida, Thidarut Boonmars, Angela Cadavid Restrepo, Mary Lorraine Sagarang Mationg, Archie C. A. Clements, Kinley Wangdi, Banchob Sripa, Darren J. Gray

**Affiliations:** 1 Department of Parasitology, Faculty of Medicine, Khon Kaen University, Khon Kaen, Thailand; 2 Office of Diseases Prevention and Control 4 Saraburi, Ministry of Public Health, Saraburi, Thailand; 3 Faculty of Public Health, Khon Kaen University, Khon Kaen, Thailand; 4 Faculty of Science and Technology, Sakon Nakhon Rajabhat University, Sakon Nakhon, Thailand; 5 Sirindhorn College of Public Health Suphanburi, Faculty of Public Health and Allied Health Sciences, Praboromarajchanok Institute, Suphanburi, Thailand; 6 Division of General Communicable Diseases, Department of Disease Control, Ministry of Public Health, Nonthaburi, Thailand; 7 Department of Mathematics, Geospatial Engineering and Innovation Center, King Mongkut’s University of Technology Thonburi, Bangkok, Thailand; 8 School of Public Health, Faculty of Medicine, The University of Queensland, Brisbane, Australia; 9 QIMR Berghofer Medical Research Institute, Brisbane, QLD Australia; 10 National Centre for Epidemiology and Population Health (NCEPH), Australian National University, Acton, ACT Australia; 11 School of Biological Sciences, Queen’s University Belfast, Belfast, UK; 12 HEAL Global Research Centre, Health Research Institute, Faculty of Health, University of Canberra, Bruce, ACT Australia; 13 Department of Tropical Medicine, Faculty of Medicine, Khon Kaen University, Khon Kaen, Thailand

**Keywords:** *Opisthorchis viverrini*, soil-transmitted helminths, Bayesian geostatistical model, Thailand, Diseases, Ecology, Ecology, Medical research, Risk factors

## Abstract

*Opisthorchis viverrini* and soil-transmitted helminths (STH) remain major public health challenges in Thailand due to their widespread distribution. This study aimed to map and predict the prevalence of *O. viverrini* and STH infections across Thailand and to identify key environmental, climatic, and socio-economic factors influencing their geographical distribution. Bayesian geostatistical logistic regression models were fitted to national survey data to estimate infection risk and generate high-resolution spatial predictions. Demographic (sex, age), environmental (land surface temperature, vegetation indices, altitude), climatic (minimum and maximum temperature), and socio-economic (nighttime light intensity) variables were included as covariates in the models, and their associations with infection risk were quantified using posterior odds ratios and 95% Bayesian credible intervals (95% CrI). *Opisthorchis viverrini* infection was significantly associated with sex and age. Males had higher odds of infection than females (OR: 1.57; 95% CrI: 1.24–1.97). Individuals aged 25–59 years (OR: 4.75; 95% CrI: 3.22–7.21) and ≥ 60 years (OR: 4.69; 95% CrI: 3.10–7.29) had similarly elevated risks compared with those < 25 years. Minimum temperature (TMIN) was significantly negatively associated with infection risk. For each 1 °C increase in TMIN, the probability of *O. viverrini* infection decreased by 33% (OR = 0.67, 95% CrI: 0.49–0.88). Hookworm infection was more common among males (OR: 1.65; 95% CrI: 1.42–1.93) and individuals aged 25–59 years (OR: 1.86; 95% CrI: 1.52–2.28). For *Ascaris lumbricoides*, infection risk was lower in individuals aged 25–59 years (OR: 0.42; 95% CrI: 0.18–0.98) and negatively associated with land surface temperature (OR: 0.66; 95% CrI: 0.39–0.98) and nighttime light intensity (OR: 0.97; 95% CrI: 0.93–0.99). No statistically significant associations were detected for *Trichuris trichiura*. Spatial predictions showed that *O. viverrini* was concentrated in northeastern Thailand, whereas STH infections were most prevalent in the south. Distinct spatial heterogeneity was observed in the distribution of *O. viverrini* and STH infections in Thailand. Demographic factors were consistently associated with *O. viverrini* and hookworm infection, whereas selected environmental and socio-economic correlates were associated with *A. lumbricoides*. These findings support geographically targeted surveillance and control strategies focused on high-burden areas and vulnerable populations.

**Supplementary Information:**

The online version contains supplementary material available at 10.1038/s41598-026-55420-0.

## Background

 Neglected tropical diseases (NTDs) remain a significant public health challenge, particularly in regions with limited healthcare access and poor sanitation infrastructure^1^. Among these, opisthorchiasis and soil-transmitted helminths (STH) infections are major concerns in many parts of the world, contributing to substantial morbidity and economic burden^2^. In Thailand, the concurrent prevalence of these parasitic infections underscores the need for a comprehensive understanding of their spatial distribution, risk factors, and transmission dynamics^3–6^.

*Opisthorchis viverrini*-associated opisthorchiasis is a significant food-borne trematodiasis in the Greater Mekong Subregion^7^. This liver fluke is endemic to Thailand, Lao People’s Democratic Republic (Lao PDR), Cambodia, Vietnam, and Myanmar, with an estimated 10 million people infected in Thailand and Lao PDR^7,8^. The highest burden is in northern and northeastern Thailand, where approximately 8 million people are affected^8^. Chronic *O. viverrini* infections cause bile duct inflammation and oxidative damage, increasing the risk of cholangiocarcinoma (CCA), a highly fatal liver cancer^9^. The primary mode of transmission is the consumption of raw or undercooked cyprinid fish harboring *O. viverrini* metacercariae. The fluke’s life cycle depends on the presence of *Bithynia* snails, which serve as the first intermediate host, and whose distribution is strongly influenced by environmental factors such as water quality, temperature, and vegetation—linking ecological conditions directly to transmission dynamics of the parasite^8,10,11^.

STH infections affect over 1.5 billion people globally, or approximately 24% of the world’s population^12^. These infections are prevalent in tropical and subtropical regions, including sub-Saharan Africa, China, South America, and Asia, where inadequate sanitation facilitates the transmission of helminth eggs and larvae via contaminated soil^12^. STH infections contribute significantly to malnutrition, anemia, impaired cognitive development, and reduced productivity. Severe cases can lead to intestinal obstruction, requiring urgent surgical intervention. According to the World Health Organization (WHO), high transmission zones affect approximately 260 million preschool-age children, 654 million school-age children, 108 million adolescent girls, and 138.8 million pregnant/lactating women^13^. Around 70% of STH infections globally occur in Asia, emphasizing the need for region-specific control strategies^2^.

*Opisthorchis viverrini* and STH remain major public health concerns in Thailand, with infections widely distributed across the country^3,4^. A recent national survey in 2019 reported an overall *O. viverrini* prevalence of 2.2%, while STH infections remain substantial, with hookworms as the most common helminth at 4.5% prevalence. The survey also found that *Ascaris lumbricoides*, *Trichuris trichiura*, and *Strongyloides stercoralis* contributed to the overall helminth burden with prevalence rates of 0.2%, 0.7%, and 0.2%, respectively^4^. Although prevalence has declined compared to earlier decades^14^, their persistence and wide geographic distribution call for improved spatial risk assessment to support control programs.

Accurate delineation of the geographical distribution of *O. viverrini* and STH infections, coupled with the identification of location-specific environmental, climatic, and socioeconomic factors influencing transmission, is crucial for effective control interventions. Traditional survey-based mapping approaches may not provide a comprehensive understanding of infection risk distribution. Bayesian model-based geostatistics offers a robust analytical framework to address these limitations, allowing for high-resolution spatial predictions and uncertainty quantification in prevalence estimates^15–18^. Predictive risk maps generated from these models have been shown to inform targeted treatment campaigns, enhance surveillance strategies, and support resource allocation in control programmes^19,20^. For example, spatial mapping of schistosomiasis in Tanzania enabled more precise targeting of mass-treatment efforts and highlighted areas needing further data collection^19^. In Brazil, STH risk maps facilitated identification of priority areas for intervention^21^, and in Côte d’Ivoire, risk profiling informed estimates of chemotherapy needs, enhancing the planning of school-based deworming campaigns^22^.

Bayesian geostatistical modelling is now widely recognised as a powerful tool in the spatial epidemiology of helminth infections including opisthorchiasis, STH infections, and schistosomiasis^23–26^. Its popularity stems from the ability to explicitly account for spatial autocorrelation, handle hierarchical data structures, and generate fully probabilistic risk predictions that incorporate uncertainty. In Southeast Asia, for example, Bayesian geostatistical models have been used to produce high-resolution maps of opisthorchiasis and STH infections at both point and area levels, providing valuable evidence to guide targeted control strategies^16,25–27^. Similar approaches have been applied to the mapping of malaria, dengue, and schistosomiasis, where the use of zero-inflated likelihoods has improved predictive performance in settings with many zero observations^23,24,28–30^.

In this study, we focused on key covariates known to influence helminth transmission, including the normalized difference vegetation index (NDVI) and normalized difference water index (NDWI) to reflect habitat suitability for intermediate hosts; monthly maximum temperature (TMAX) and minimum temperature (TMIN) to account for temperature effects on parasite development and human behavior; altitude (ALT) to capture ecological gradients; and nighttime lights (NTL) as a proxy for human settlement and socioeconomic activity. Incorporating these covariates in Bayesian geostatistical models enables high-resolution spatial prediction while quantifying uncertainty in prevalence estimates. Unlike previous spatial studies in Thailand, which were generally restricted to localized endemic settings, selected provinces, or single helminth species^3,16,26^, the present study utilized the most recent nationwide helminth survey conducted using a standardized sampling framework across Thailand. This study provides the first nationwide Bayesian geostatistical assessment integrating multiple helminth infections with demographic, environmental, climatic, and socio-economic covariates at high spatial resolution. In addition, the modelling framework enabled quantification of uncertainty in predicted prevalence across unsampled areas, which is important for geographically targeted surveillance, resource allocation, and disease control planning. Therefore, this study aimed to examine the associations between demographic, environmental, climatic, and socio-economic covariates and *O. viverrini* and STH infections in Thailand, and to generate predictive spatial risk maps using Bayesian geostatistical logistic regression models.

## Methods

### Study area

**Fig. 1:**
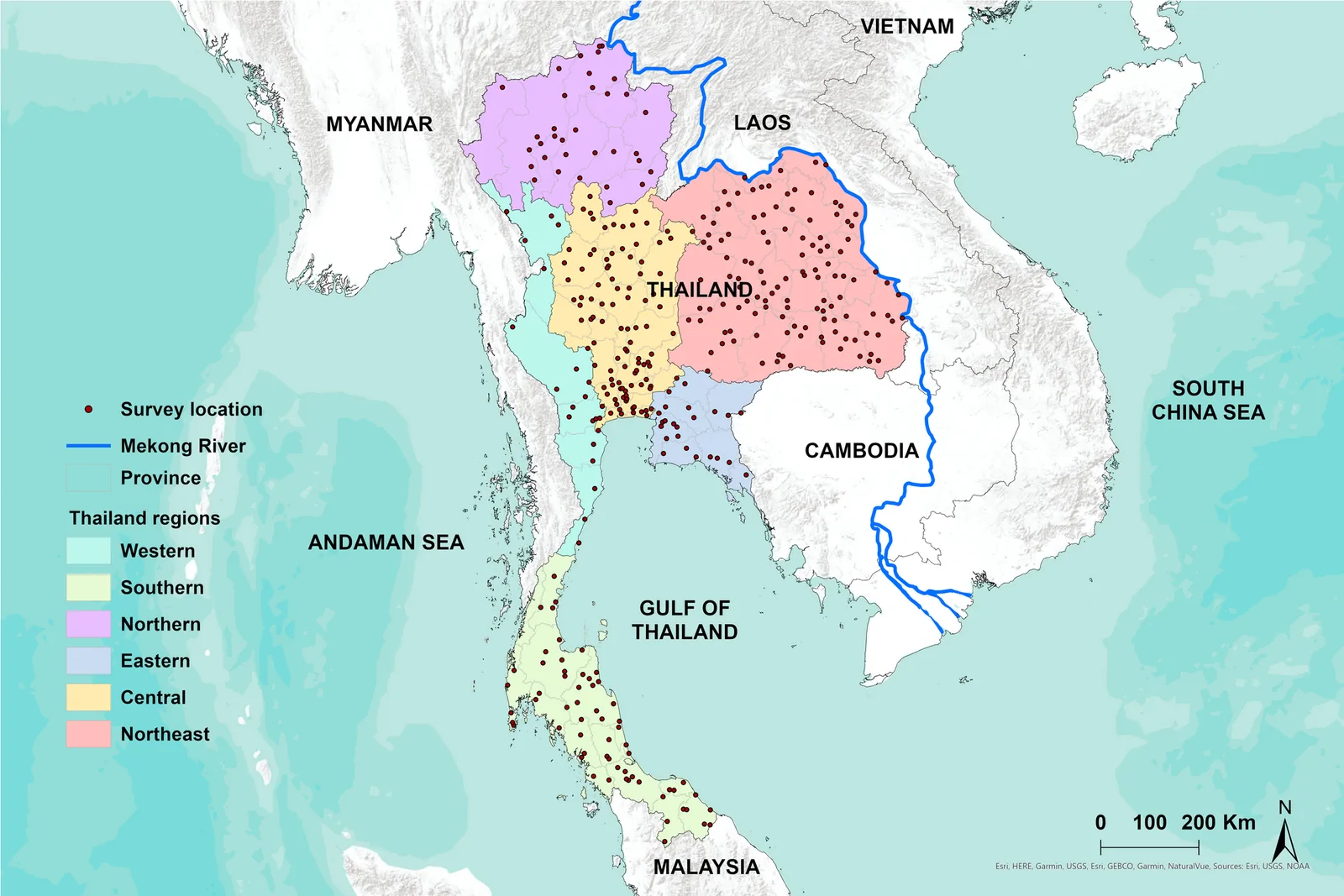
Geographical regions and survey locations of *Opisthorchis viverrini* and soil-transmitted helminths in six regions of Thailand between January and July in 2019

Situated in Southeast Asia, Thailand is characterized by a tropical climate within the geographical coordinates of longitudes 97° 22′ and 105° 37′ E and latitudes 5° 37′ and 20° 27′ N. As of 2021, the total population of the country reached 71.6 million, with approximately 34 million individuals (48.24%) residing in rural areas. Geographically, Thailand is divided into six regions: Northern, Northeastern, Central, Western, Eastern, and the Southern region (Fig. 1). The administrative framework of Thailand exhibits a hierarchical structure, encompassing 76 provinces and one distinctive special administrative area or capital, Bangkok. This administrative configuration further includes 928 districts, 7,256 sub-districts, and 75,668 villages (https://dopa.go.th/). Thailand experiences three distinct seasons, as characterized by the Thai Meteorological Department (https://www.tmd.go.th/): the hot season (mid-February to mid-May), the rainy season (mid-May to mid-October), and the cool season (mid-October to mid-February).

### Demographic and parasitology data

The data used in this study were obtained from the latest cross-sectional nationwide survey on helminths conducted between January and July in 2019. This comprehensive survey was undertaken by the Thailand Ministry of Public Health. In-depth details of the survey methodology have been elucidated by Wattawong et al. in 2021^4^. In summary, the survey encompassed a healthy population spanning from 6 months to over 60 years. Employing a multi-stage cluster random sampling design with probability proportional to size, the survey was conducted in 361 locations from all 76 provinces in Thailand (Fig. 1). This survey aimed to assess the prevalence of helminth infections and demographic data. A total of 16,187 stool samples were collected from the participants. Stool examination utilized the formalin-ethyl acetate concentration technique and modified Kato-Katz technique, with experienced parasitologists identifying parasite eggs/larva under light microscope. Demographic information, comprising age and sex, was collected from all study participants through a questionnaire. The geographical coordinates of surveyed locations were sourced from the OpenStreetMap database in the QGIS software (Quantum, 2023).

### Environmental, climatic, and socio-economic variables

The data used in this study are shown in Table 1. To account for potential annual fluctuations in environmental, climatic, and socio-economic conditions while still capturing relatively recent data, we downloaded data for Thailand spanning a 5-year period from 2015 to 2019. This approach enabled us to compute the mean values for these variables over this period, providing a robust average that smooths out short-term variability. These variables were extracted from various satellite images and composite products. JavaScript code was utilized for Google Earth Engine (GEE), a widely adopted tool in diverse fields of earth science and environmental science^31,32^. Land surface temperature (LST) was obtained from the MYD11A1 V6.1 product, providing both daytime and night-time surface temperature bands along with their corresponding quality indicator layers. The normalized difference vegetation index (NDVI) and normalized difference water index (NDWI) were derived from the Sentinel-2 multi-spectral imaging mission and MODIS surface reflectance composites, respectively^33^. Monthly maximum temperature (TMAX), monthly minimum temperature (TMIN), and altitude (ALT) data were acquired from the WorldClim database. Nighttime light (NTL) data were obtained from the Visible Infrared Imaging Radiometer Suite (VIIRS) Day/Night Band (DNB) monthly average radiance composite images^34^. Thailand administrative boundaries were obtained from DIVA-GIS.

**Table 1:** Source and characteristics of the environmental, climatic, and socio-economic variables used to model *Opisthorchis viverrini* and soil-transmitted helminth infection risk in Thailand

Variables	Units	Details	Resolution	Source	Dataset name
Land surface temperature (LST)	°C	5-year average (2015–2019)	1000 m	Google Earth Engine	MODIS/061/MYD11A1
Normalized difference vegetation index (NDVI)	Index	5-year average (2015–2019)	10 m	Google Earth Engine	COPERNICUS/S2
Normalized difference water index (NDWI)	Index	5-year average (2015–2019)	463.313 m	Google Earth Engine	MODIS/MOD09GA_006_NDWI
Minimum temperature (TMIN)	°C	5-year average (2015–2019)	21 km^2^	WorldClim	
Maximum temperature (TMAX)	°C	5-year average (2015–2019)	21 km^2^	WorldClim	
Altitude (ALT)	masl	The height of an object or point in relation to sea level or ground level	21 km^2^	WorldClim	
Nighttime light (NTL)	nW/cm^2^/sr	5-year average (2015–2019)	463.83 m	Google Earth Engine	NOAA/VIIRS/DNB/MONTHLY_V1/VCMSLCFG
Boundary data		Thailand’s sub-district, district, and provincial boundary data		DIVA-GIS	

masl: metres above sea level.

WorldClim: https://www.worldclim.org.

DIVA-GIS: https://www.diva-gis.org/.

For this study, we utilized the point sampling tool in the QGIS software to extract values of environmental, climatic, and socio-economic raster data from their respective geospatial layers at the surveyed locations. The extracted values represent the 5-year average for the period spanning from 2015 to 2019. To predict and map the prevalence of *O. viverrini* and STH for the entire territory (including unsampled locations), we generated a grid with dimensions of 10 × 10 km. Considering the nationwide scale of Thailand and the spatial distribution of the survey locations, this grid resolution was selected to provide adequate spatial detail for capturing broad geographical patterns while maintaining computational efficiency and minimizing overinterpretation in areas with sparse sampling coverage. Similar spatial prediction grid approaches have been applied in previous Bayesian geostatistical studies of helminth infections^19,35^. Values of the spatial covariates were extracted at each grid node for spatial prediction^16,35^. At the nodes of this grid, values corresponding to spatial covariates were extracted^16,35^.

### Variable selection

Environmental, climatic, and socio-economic variables were assessed for multicollinearity utilizing STATA version 14 software (StataCorp. 2015. Stata Statistical Software: Release 14. College Station, TX: StataCorp LP). Univariate logistic regression models were constructed to evaluate the association between the observed number of helminth infections in each surveyed location and individual environmental, climatic, and socio-economic variables. Any variables with Wald’s P value > 0.2 were excluded from further analyses. The backward stepwise multivariable logistic regression analysis was executed with the remaining variables, utilizing P value ≤ 0.05 to determine included variables and Wald’s *P* > 0.1 for exclusion^19^. The explanatory variables, encompassing sex, age, LST, NDVI, ALT, TMIN, TMAX, and NTL, demonstrated statistical significance in relation to *O. viverrini* and STH infections. Variables with a variance inflation factor (VIF) exceeding 10 were excluded from subsequent model analyses^36^. The Bayesian geostatistical model for opisthorchiasis included sex, age, NDVI, TMIN, and NTL. For hookworm infections, the final model incorporated sex, age, LST, NDVI, and TMIN. The model for trichuriasis included sex, age, LST, NDVI, and TMAX. Finally, for ascariasis, the selected variables for the final model were sex, age, LST, ALT, and NTL. Nonlinear relationships between each helminth infection and the environmental, climatic, and socio-economic variables were explored by including quadratic terms in the models.

### Spatial Bayesian geostatistics models

Multivariable geostatistical logistic regression models were separately constructed for infection with *O. viverrini*, hookworms, *T. trichiura*, and *A. lumbricoides* using WinBUGS version 1.4.3 (Medical Research Council, Cambridge, UK). These models incorporated covariates identified in the non-spatial analyses. For each helminthic infection, multiple models were fitted.

The unstructured (non-spatial) model included only fixed effects for the explanatory variables and did not account for spatial dependence. The covariates comprised age, categorized into three groups (< 25 years, 25–59 years, and ≥ 60 years, with < 25 years as the reference category), based on previous helminth epidemiological studies in Thailand^4^; sex (male and female); and selected environmental, climatic, and socio-economic variables included to assess their associations with infection risk. The spatially structured model included the same fixed effects as the non-spatial model, together with a spatially structured random effect to account for spatial autocorrelation. Model selection was based on the deviance information criterion (DIC), with lower values indicating better model fit. The statistical significance of covariates was assessed using the 95% Bayesian credible interval (95% CrI) of the odds ratio (OR), where exclusion of 1 indicated a significant association. For each helminthic infection, the best-fit model included a spatially structured random effect. Assuming a Bernoulli-distributed dependent variable, *Y*_*ij*_, representing the infection status of an individual *i* in location *j* (0 = non-infected, 1 = infected), the model structure was defined as follows:

$$\:{Y}_{ij}\:\sim\:Bern\left({p}_{ij}\right)\:$$

Risk factors were then modelled on the logit scale as:

$$\:\mathrm{l}\mathrm{o}\mathrm{g}\mathrm{i}\mathrm{t}\:\left({p}_{ij}\right)=\:\alpha_{\varepsilon}\:+{{\gamma\:\:\mathsf{x}\:\mathrm{s}\mathrm{e}\mathrm{x}}_{\:}}_{i}+{\updelta\:}\:\mathsf{x}\:age+{\sum\:}_{z=1}^{z}{\beta\:}_{z}\:{\mathsf{x}\:{\uplambda\:}}_{zj}+\:{s}_{j}$$

where *α*_*ε*_ represents the intercept, γ and δ are the coefficient for sex and age, respectively, β is a matrix of coefficients for z, *λ* is a matrix of climatic and environmental variables z, and *s*_*j*_ denotes the geostatistical random effect. The correlation structure of the geostatistical random effect was modeled using an exponential function of the distance between locations:

$$\:f\left({d}_{ab};\phi\:\right)=\mathrm{e}\mathrm{x}\mathrm{p}[-\phi\:{d}_{ab}]$$

where *d*_*ab*_ represents the distances between pairs of locations *a* and *b*, and *ϕ* defines the rate at which spatial correlation decreases with distance. A normal distribution was assigned to the intercept and the coefficients, which a mean of 0 and a precision (inverse variance) of 1 × 10^–3^. The prior for *ϕ* followed a uniform distribution with bounds set at 0.01 and 100, ensuring that that the correlation remained below 0.5 at the maximum distance between survey sites. A non-informative gamma distribution (shape and scale parameters = 0.001, 0.001) was used for the precision parameter.

Markov chain Monte Carlo (MCMC) simulation was used to estimate the posterior means and compute 95% CrI. An initial burn-in of 1,000 iterations was discarded. Subsequently, 100,000 MCMC iterations were run using a single chain and assessed for convergence through visual inspection of history and density plots, together with autocorrelation analysis of the model parameters. To reduce autocorrelation, every 10th iteration was retained, resulting in 10,000 posterior samples for inference. The rate of spatial correlation decay with distance () and the variance of the spatially structured random effect (σ²) were also estimated. A regular 10 km×10 km grid was overlaid over the entire study territory to define the prediction locations. The *spatial.unipred* function in WinBUGS was used to estimate the risk of infection at the prediction locations. The function uses the model equation at each non-sampled location using the covariates values extracted for those locations and the distances between the prediction locations and the surveyed locations. Maps representing the posterior medians and the lower and upper limits of the 95% CrI were created in ArcGIS program (ESRI, Redlands, CA, USA) to visualize predicted infection prevalence and associated uncertainty. The uncertainty maps are provided in the Supplementary Information (Figs. S2–S5).

## Results

### Spatial distribution of *O. viverrini* and STH infections in Thailand

**Fig. 2:**
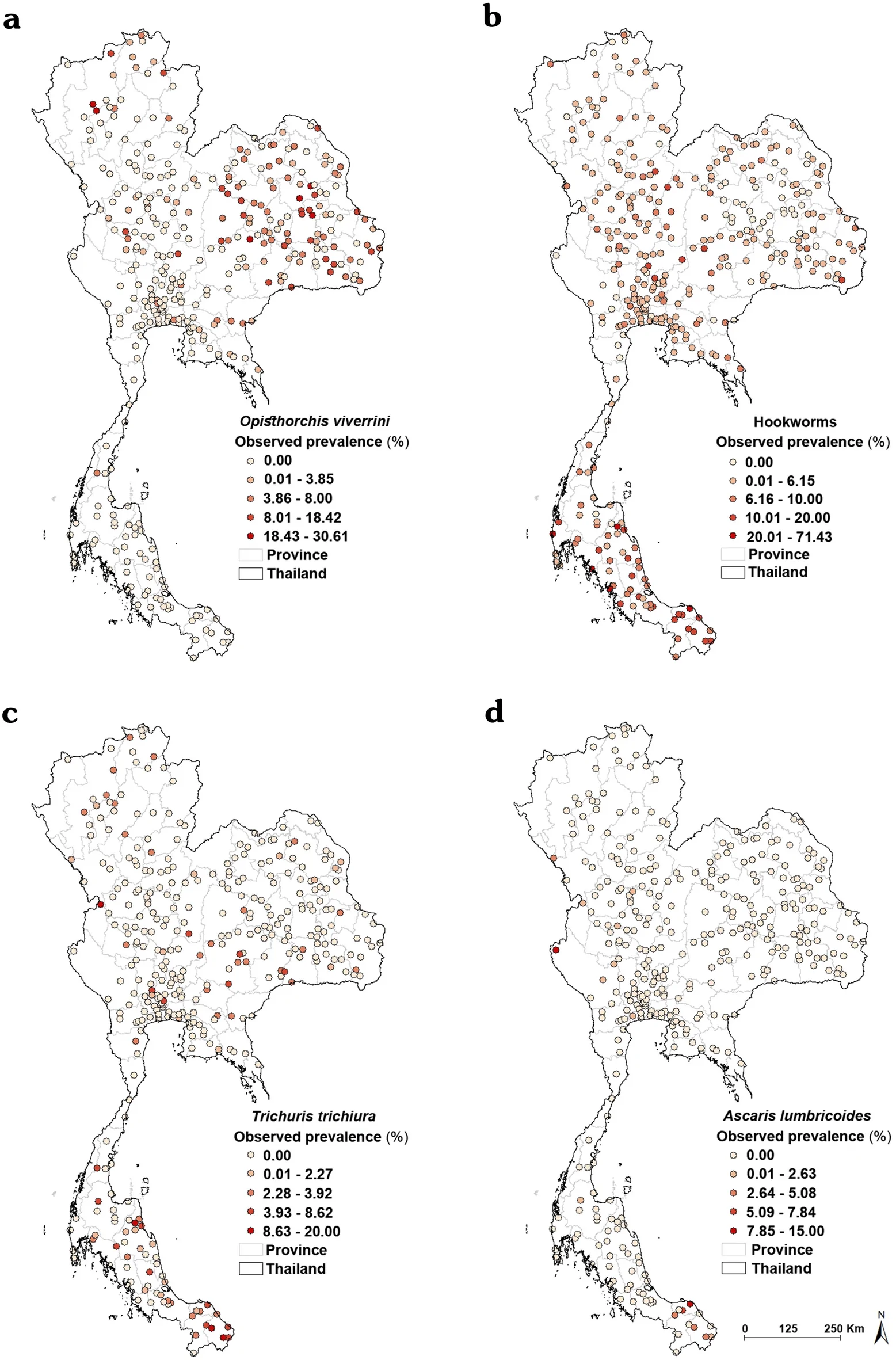
The spatial distribution of prevalence rates of *Opisthorchis viverrini* (**a**), hookworms (**b**), *Trichuris trichiura* (**c**), and *Ascaris lumbricoides* (**d**) in Thailand

The observed spatial distributions of *O. viverrini* and each STH infection prevalence in the surveyed locations are shown in Fig. 2. *Opisthorchis viverrini* was identified in the stool samples from participants in 119 of the 361 surveyed locations with prevalence ranging from 1.39% to 30.61% (Fig. 2a). Hookworms exhibited the broadest geographical distribution, detected in 289 locations (79.5% of sites) with a prevalence range of 1.39% to 71.43% (Fig. 2b). *Trichuris trichiura* was found in 73 locations (20.2% of sites), with prevalence ranging from 1.49% to 20.00% (Fig. 2c). *Ascaris lumbricoides* was detected in 15 locations (4.15% of sites), with prevalence ranging from 1.56% to 15.00% (Fig. 2d). Maps of the spatial distribution of environmental, climatic, and socio-economic covariates used in Bayesian variable selection are provided in Additional file 1: Fig. S1.

### Risk factors associated with* O. viverrini* and STH infections

#### Opisthorchiasis

The best-fitting model for *O. viverrini* infection, as indicated by the lowest DIC (1361.70), was Model II when compared with model I (Table 2 and Table S1). This model demonstrated a statistically significant positive association between *O. viverrini* infection and both sex and age. Males had a 57% higher likelihood of infection compared to females (OR = 1.57, 95% CrI: 1.24–1.97). Individuals aged 25–59 years were nearly five times more likely to be infected than those under 25 years (OR = 4.75, 95% CrI: 3.22–7.21), while those aged ≥ 60 years had a similarly increased risk (OR = 4.69, 95% CrI: 3.10–7.29). Environmental factors also played a role, with TMIN showing a significant negative association with infection risk. For each 1 °C increase in TMIN, the probability of *O. viverrini* infection decreased by 33% (OR = 0.67, 95% CrI: 0.49–0.88).

**Table 2:** Bayesian geostatistical logistic regression models of *Opisthorchis viverrini*, hookworms, *Trichuris trichiura*, and *Ascaris lumbricoides* in Thailand.

Variables	*Opisthorchis viverrini*	Hookworms	*Trichuris trichiura*	*Ascaris lumbricoides*
	OR (97.5% CrI)	OR (97.5% CrI)	OR (97.5% CrI)	OR (97.5% CrI)
Intercept (log odds scale)^a^	-6.53 (-7.35–5.83)	-3.30 (-3.69 - -2.83)	-6.09 (-6.76 - -5.49)	-11.44 (-16.83 - -8.07)
Male (vs. female)	1.57 (1.24–1.97)*	1.65 (1.42–1.93)*	0.97 (0.66–1.40)	1.18 (0.57–2.46)
25–59 years old (vs. age < 25 years old)	4.75 (3.22–7.21)*	1.86 (1.52–2.28)*	1.04 (0.67–1.64)	0.42 (0.18–0.98)*
Age ≥ 60 years (vs. age < 25 years old)	4.69 (3.10–7.29)*	1.41 (1.12–1.78)*	1.16 (0.71–1.91)	0.76 (0.31–1.84)
LST	-	0.96 (0.91–1.02)	0.84 (0.70–1.09)	0.66 (0.39–0.98)*
NDVI	0.01 (0.00–0.25)*	0.95 (0.31–3.33)	0.12 (0.00–3.67)	-
ALT	-	-	-	1.00 (0.99–1.00)
TMIN	0.67 (0.49–0.88)*	1.13 (0.95–1.30)	-	-
TMAX		-	0.82 (0.55–1.12)	-
NTL	1.00 (0.99–1.00)	-	-	0.97* (0.93–0.99)
ϕ (rate of decay of spatial correlation)	10.85 (0.98–83.58)	3.79 (0.32–47.83)	34.34 (1.28–95.83)	57.33 (13.86–97.77)
σ^2^ (variance of spatial random effect)	0.31 (0.15 0.49)	2.60 (0.99 4.79)	0.56 (0.31–0.94)	10.82 (0.03–0.30)
DIC	1361.70	2773.19	775.10	199.22

* indicates statistically significant at *p* < 0.05, ^a^Coefficient of posterior mean *LST* Land surface temperature *NDVI* Normalized difference vegetation index *ALT* Altitude *TMIN* Minimum temperature *TMAX* Maximum temperature *NTL* Nighttime lights: *Bayes. CrI* Bayesian credible interval, *OR* odds ratio *DIC* Deviance information criterion.

The spatial correlation decay parameter for *O. viverrini* infection was estimated at 10.85 (95% CrI: 0.98–83.58), suggesting a moderate rate of spatial autocorrelation decay. This indicates that the probability of infection was higher in geographically proximate areas, with the spatial dependency decreasing as distance increased. The variance of the spatial random effect was 0.31 (95% CrI: 0.15–0.49), highlighting spatial heterogeneity in infection risk across study locations (Table 2).

#### Hookworm infection

The best-fitting model for hookworm infection, as indicated by the DIC value of 2773.19, was Model II when compared with model I (Table 2 and Table S2). A statistically significant positive association was found between hookworm infection and both sex and age. Males exhibited a 65% higher likelihood of being infected with hookworm compared to females (OR = 1.65, 95% CrI: 1.42–1.93). Individuals aged 25–59 years and ≥ 60 years were 86% (OR = 1.86, 95% CrI: 1.52–2.28) and 41% (OR = 1.41, 95% CrI: 1.12–1.78) more likely to be infected, respectively, compared to those under 25 years.

The analysis revealed a negative correlation between LST and NDVI with hookworm infection, although this correlation did not reach statistical significance. Additionally, the spatial correlation decay parameter for hookworm infection was estimated at 3.79 (95% CrI: 0.32–47.83), indicating a relatively low to moderate spatial autocorrelation effect. The variance of the spatial random effect for hookworm infection was 2.60 (95% CrI: 0.99–4.79), suggesting substantial spatial variability in infection risk across surveyed locations (Table 2).

#### Trichuriasis

For *T. trichiura*, the model II with the lowest DIC value of 775.10, was identified as the best fit model when compared with model I (Table 2 and Table S3). No statistically significant associations were detected between *T. trichiura* infection and sex or age, (OR for males vs. females = 0.97, 95% CrI: 0.66–1.40; OR for age 25–59 years vs. age < 25 years = 1.04, 95% CrI: 0.67–1.64; OR for age ≥ 60 years vs. age < 25 years = 1.16, 95% CrI: 0.71–1.91).

The spatial correlation decay parameter for *T. trichiura* infection was estimated at 34.34 (95% CrI: 1.28–95.83), indicating a moderately high level of spatial autocorrelation. The variance of the spatial random effect was 0.56 (95% CrI: 0.31–0.94), further suggesting significant spatial heterogeneity in *T. trichiura* infection risk (Table 2).

#### Ascariasis

For *A. lumbricoides* infection, Model II (DIC = 199.22) was identified as the best-fitting model compared with Model I (Table 2 and Table S4). No statistically significant association was detected between ascariasis and sex (OR for males vs. females = 1.18, 95% CrI: 0.57–2.46). In contrast, age showed a significant association with infection risk. Individuals aged 25–59 years had a significantly lower likelihood of infection compared with those under 25 years of age (OR = 0.42, 95% CrI: 0.18–0.98). Although individuals aged ≥ 60 years had a higher estimated odds of infection, (OR = 0.76, 95% CrI: 0.31–1.84), this association was not statistically significant.

The presence of LST and NTL were significantly associated with ascariasis. For each 1 °C increase in LST, the probability of ascariasis prevalence decreased significantly by 34% (OR = 0.66; 95% CrI: 0.39–0.98). Similarly, with each unit increase in NTL, commonly used as a proxy for urbanization and human economic activity, the probability of ascariasis prevalence decreased by 3% (OR = 0.97; 95% CrI: 0.93–0.99).

The rate of decay of spatial correlation of ascariasis infection was 57.33 (95% CrI: 13.86–97.77), indicating that the spatial correlation for ascariasis infection primarily occurs over short distances, with higher infection risks in neighboring or proximate areas. These findings suggest strong spatial clustering of ascariasis infection (Table 2).

### Predicted prevalence

**Fig. 3:**
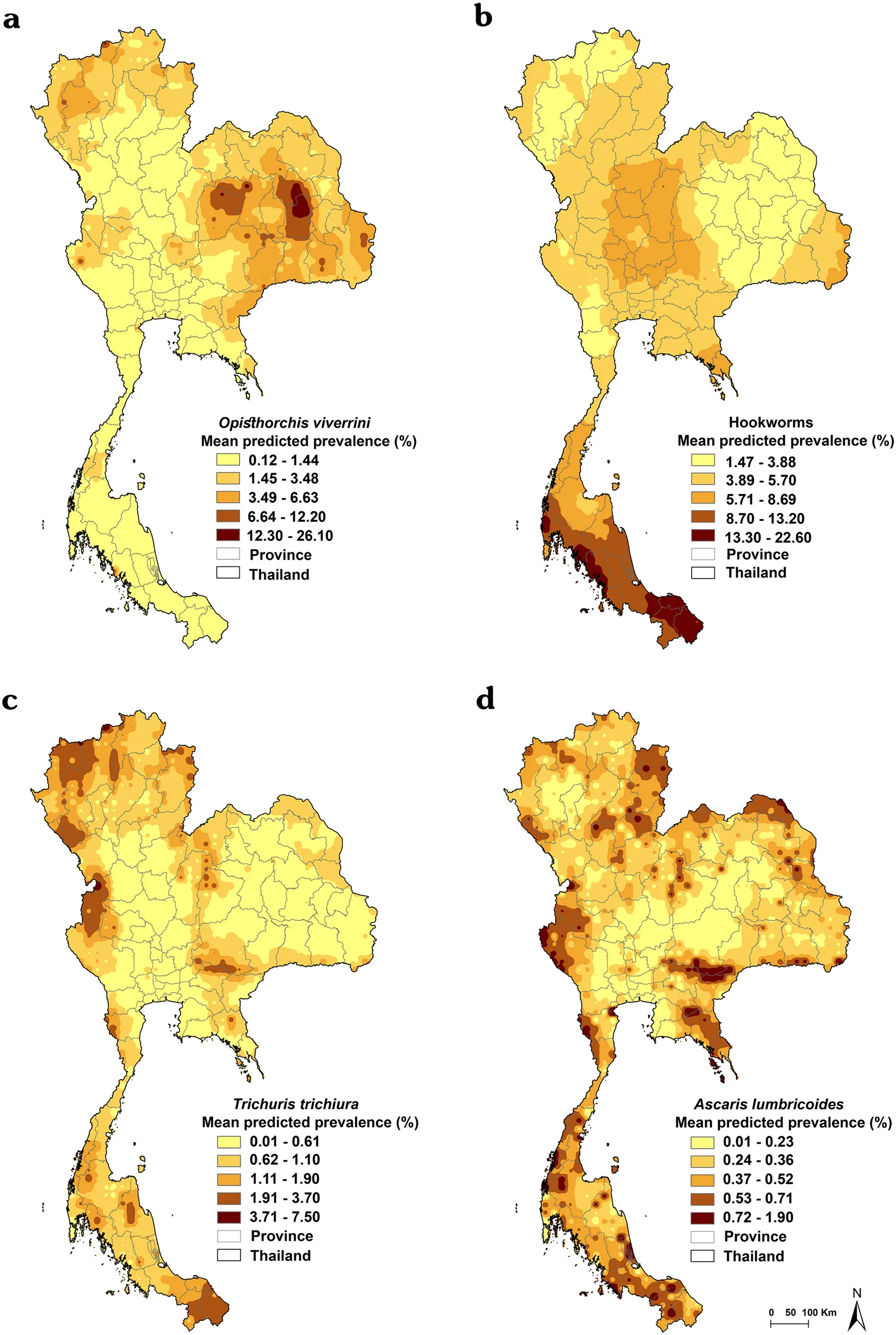
Predicted mean prevalence of *Opisthorchis viverrini* (**a**), hookworms (**b**), *Trichuris trichiura* (**c**), and *Ascaris lumbricoides* (**d**) in Thailand. Dark brown indicates areas with the highest predicted prevalence, while light yellow indicates areas with the lowest predicted prevalence

The predicted prevalence analysis indicated the highest predicted prevalence (dark brown) of the *O. viverrini* infection in northeast Thailand, specifically in the provinces of Chaiyaphum, Khon Kaen, Roi Et, and Kalasin. Conversely, the lowest-risk areas (light yellow) for *O. viverrini* prevalence were identified in the central, northern, western, and southern regions of Thailand (Fig. 3a).

For STH, the areas with the highest predicted prevalence (dark brown) were observed in the southern part of the country, particularly in the provinces of Songkhla, Pattani, Yala, and Narathiwat for hookworm (Fig. 3b). Trichuriasis was primarily concentrated in the southern regions of Narathiwat, Yala, and Pattani, as well as the northern region, including Mae Hong Son and Chiang Mai provinces, and the western region, including Tak Province (Fig. 3c). For ascariasis, the highest predicted prevalence areas were found in the southern provinces of Yala, Pattani, Songkhla, and Satun; the western region, including Tak and Kanchanaburi provinces; the northern region, encompassing Nan Province; the northeastern region, represented by Bueng Kan; and the eastern region, including Sa Kaeo, Chanthaburi, and Trat provinces (Fig. 3d). In contrast, areas with the lowest predicted prevalence (light yellow) for all STH were observed in other parts of the country (Fig. 3). Uncertainty maps showing the lower and upper limits of the 95% Bayesian credible intervals for predicted prevalence are presented in Supplementary Figs. S2–S5.

## Discussion

This nationwide Bayesian geostatistical analysis demonstrated substantial spatial heterogeneity in the distribution of *O. viverrini* and STH infections across Thailand. The predicted prevalence of *O. viverrini* remained highest in northeastern Thailand, whereas STH infections, particularly hookworms, were concentrated in southern regions. Demographic characteristics, especially sex and age, were consistently associated with *O. viverrini* transmission, while environmental factors, including NDVI and TMIN, contributed to spatial heterogeneity in prevalence. For hookworms, transmission was associated primarily with demographic characteristics, particularly age and sex. *Trichuris trichiura* showed low prevalence and no significant associations with either demographic or environmental variables, although spatial predictions still highlighted localized areas of infection. In contrast, *Ascaris lumbricoides* was influenced by both demographics and environmental variables, including LST and NTL.

Spatial prediction from Bayesian models revealed distinct geographic patterns, *O. viverrini* prevalence was highest in northeastern Thailand, while STH, including hookworms, *T. trichiura*, and *A. lumbricoides*, predominated in southern regions. In our study, Bayesian geostatistical modeling allowed us to quantify spatial heterogeneity and uncertainty in the prevalence of *O. viverrini* and STH infections, providing predicted prevalence in unsampled districts across Thailand. This approach revealed location-specific demographic and environmental drivers, such as age, sex, NDVI, TMIN, LST, and NTL, that were not consistently identified in conventional multivariate analyses, demonstrating the added value of spatial models for informing targeted interventions.

Our findings are consistent with previous reports emphasizing that environmental conditions play an important role in the transmission. *Opisthorchis viverrini* and STH infections are influenced by various environmental factors including vegetation, land cover, elevation, temperature, and water availability, all of which contribute to the potential exposure of individuals to such infections^37–39^. By integrating these variables into Bayesian spatial models, we were able to quantify their relative contributions and capture spatial heterogeneity, which traditional multivariate analyses cannot fully resolve.

Over the past four decades, the liver fluke control program has been continuously implemented, with its intensity fluctuating in accordance with government policies and national campaigns targeting the elimination of *O. viverrini* and CCA. The nationwide survey indicated a noteworthy decrease in the prevalence of *O. viverrini* infection. However, policy makers have focused much more on the liver fluke than on STH and previous data showed that STH infections were the major helminthiasis with the highest prevalence in this study in the South of Thailand^4,8^.

We observed a higher risk of *O. viverrini* and STH infections in males. This aligns with a prior study conducted in Thailand, which similarly noted an increased likelihood of *O. viverrini* in males^16^ and risk of STH infection was positively associated with males in the Philippines^35^. The increased prevalence of *O. viverrini* infection in males, as opposed to females, can be attributed to behavioral patterns such as the consumption of raw cyprinid fish, smoking habits, alcohol intake, and genetic factors^15^. Therefore, this sex-based disparity contributes to elevated rates of CCA^40^.

Our results indicated a notable association between LST and a decreased likelihood of ascariasis risk. This aligns with a similar finding in the People’s Republic of China, where LST was linked to a reduced risk of *A. lumbricoides* infection^20^. Specifically, each 1 °C increase in LST corresponded to a 34% decrease in *A. lumbricoides* prevalence (OR = 0.66; 95% CrI: 0.39–0.98), highlighting the importance of local temperature in controlling egg and larval survival.

Previous studies show that mean annual precipitation, land cover, and altitude significantly influence STH distribution in Thailand^3^. By explicitly incorporating these variables into Bayesian models, we could predict spatial variation and estimate uncertainty in areas with sparse sampling. Evidence shows rising temperatures and altered rainfall patterns in Thailand^41^, which directly affect parasite life cycles. For example, hookworms thrive within 26–30 °C and require specific moisture conditions; thus, small climatic shifts can substantially impact prevalence^42,43^. Poor sanitation, hygiene practices, and malnutrition further exacerbate transmission^44,45^. Climate change also influences NDVI variability, which serves as a proxy for vegetation and potential exposure risk to parasitic infections. Reduced NDVI, often in urbanized areas, was associated with increased *A. lumbricoides* transmission, whereas higher NDVI reflected favorable agro-climatic conditions that may reduce exposure^35,46,47^.

The transmission of STH is significantly influenced by socio-economic factors, primarily shaping people’s behavior^48^. At a smaller scale, variables such as socio-economic status, occupational exposure, behavior, and access to safe drinking water and proper sanitation facilities may play a more substantial role than natural environmental factors in explaining the spatial variation in the prevalence of STH co-infections in Luzon and the Visayas^49^. Our study showed that increased NTL was associated with a 3% decrease in the probability of ascariasis prevalence per unit increase (OR = 0.97; 95% CrI: 0.93–0.99). The negative association between NTL and *A. lumbricoides* suggests that areas with higher levels of NTL, commonly used as a proxy for urbanization and human economic activity, may be associated with environmental and socio-economic conditions less favorable for transmission. NTL may reflect underlying socio-environmental factors such as improved sanitation infrastructure, greater access to clean water, higher levels of health awareness, and reduced environmental contamination with infective eggs. These conditions are generally less favorable for the transmission of *A. lumbricoides*, which is closely linked to poor hygiene and inadequate sanitation^19,49^. However, NTL may not fully capture local variations in income inequality, sanitation quality, or household-level socio-economic conditions. Therefore, the observed association should be interpreted cautiously.

Importantly, Bayesian geostatistical models allowed us to quantify spatial heterogeneity and uncertainty in prevalence estimates, particularly identifying areas with sparse data and low-prevalence infections such as *T. trichiura* and *A. lumbricoides*. Model II, the spatially structured model, provided the best fit, highlighting the importance of accounting for spatial correlation in prevalence estimates. This spatially explicit information is valuable for prioritizing high-burden areas, targeting interventions, and allocating resources efficiently—capabilities not achievable with conventional analyses alone.

Our model-based predictive prevalence for 2019 estimated that *O. viverrini* infection remained highest in Northeast Thailand (predicted prevalence 0.12–26.10%), while STH infections were concentrated in the South (predicted prevalence 1.47–22.60%). Recent observational studies from 2020 to 2025 report *O. viverrini* prevalence ranging from 0.60 to 30.00% in the Northeast^50–53^ and STH prevalence of 2.10–10.90% in Southern provinces^54–57^. The close alignment between our predictive results and these updated observations supports the utility of our Bayesian geostatistical model. These recent data could also serve as an external validation source, allowing future studies to refine model predictions and improve risk-based targeting of interventions.

Our study further demonstrates that Bayesian geostatistical models can predict prevalence in unsampled locations and identify location-specific environmental, climatic, and socio-economic drivers, including NDVI, TMIN, LST, and NTL, which may not be consistently captured in conventional multivariate analysis, which primarily detect associations with individual-level factors such as age and sex. Consequently, these models support geographically targeted interventions, inform public health planning, and are suitable for monitoring *O. viverrini* and STH infections in Thailand and other countries facing similar disease challenges.

The conclusions derived from this study should be interpreted with awareness of certain limitations. First, concerning data and covariates, the study relied on environmental and climatic variables from publicly available datasets. Although meteorological stations exist, their spatial coverage across the country is uneven at fine resolutions. In addition, important socio-economic and behavioral risk factors—such as education, income, employment, poverty, raw fish consumption, footwear practices, and access to sanitation—were not available. The omission of these variables may limit the explanatory power of the models. Second, regarding population and survey data, district-level population counts were derived from census-based estimates, which may have led to under- or overestimation. Furthermore, the survey data used were not specifically designed for risk mapping, although they provided wide geographic coverage across Thailand. Third, from an epidemiological perspective, the very low prevalence of certain infections, particularly *T. trichiura* and *A. lumbricoides*, reduced the reliability of spatial predictions and increased parameter uncertainty. The observed variation in spatial random effects further highlights the challenges of modeling infections with sparse data. Therefore, the predicted prevalence surfaces for these infections should be interpreted cautiously, particularly in areas with sparse sampling coverage^12,17^. Future studies incorporating larger sample sizes or targeted surveys in low-prevalence areas will be important to enhance the robustness of spatial predictions for these parasites.

The major strength of this study is the use of a nationwide standardized survey dataset of *O. viverrini* and STH infections covering all regions of Thailand. While previous spatial studies in Thailand have largely focused on specific endemic areas, selected provinces, or individual helminth infections using localized datasets or single-disease approaches^3,16,26^. In contrast, this study integrated multiple helminth infections with demographic, environmental, climatic, and socio-economic variables within a Bayesian geostatistical framework. In addition, the Bayesian approach accounted for spatial uncertainty and improved prevalence estimation, particularly for infections with relatively few observed cases.

## Conclusions

This study provides a nationwide, model-based characterization of the spatial distribution of *O. viverrini* and STH infections in Thailand using Bayesian geostatistical methods. The results indicate that *O. viverrini* remains concentrated in northeastern Thailand, whereas STH infections are more prevalent in southern regions, reflecting substantial geographic heterogeneity in infection risk. By integrating spatial dependence into the modeling framework, we were able to generate smoothed prevalence surfaces and quantify associated uncertainty, particularly in areas with sparse data. Although these findings are derived from secondary survey data and should therefore be interpreted with appropriate caution, the spatial patterns identified here may support the prioritization of surveillance and control efforts toward higher-risk areas. Future studies incorporating behavioral, environmental, and longitudinal data would be valuable for improving model precision and for better understanding the drivers of transmission dynamics.

## Supplementary Information

Below is the link to the electronic supplementary material.

Supplementary Material 1

## Data Availability

The data that support the findings of this study were obtained from a nationwide parasitic disease surveillance survey conducted by the Thailand Ministry of Public Health. Due to ethical and legal restrictions related to national health data protection policies, the data are not publicly available. Access to the data may be granted upon reasonable request and subject to approval from the Thailand Ministry of Public Health.
