# Supplementary material for: Spatial patterns and risk mapping of opisthorchiasis and soil-transmitted helminth infections in Thailand using Bayesian geostatistical models
Source: Sci Rep. 2026 Jun 11;16:26594. doi: 10.1038/s41598-026-55420-0 (PMC13503900; doi:10.1038/s41598-026-55420-0)
Supplement: Supplementary file 1 [file 41598_2026_55420_MOESM1_ESM.pdf]

Supplementary Information

Additional file 1: Figures and Tables

Spatial patterns and risk mapping of opisthorchiasis and soil-transmitted helminth infections in Thailand using Bayesian geostatistical models

Khanittha Pratumchart[1], Apiporn T. Suwannatrai[1]*, Oranard Wattanawong[2], Kavin Thinkhamrop[3], Kulwadee Suwannatrai[4], Ampas Wisetmora[5], Suphansa Wichairattanagull[2], Nichaphat Kukitirat[2], Thitima Wongsaroj[6], Pariwate Varnakovida[7], Thidarut Boonmars[1], Angela Cadavid Restrepo[8], Mary Lorraine Sagarang Mationg[9,10], Archie C.A. Clements[11], Kinley Wangdi[10,l2], Banchob Sripa[13], Darren J. Gray[9]

*Corresponding author. Department of Parasitology, Faculty of Medicine, Khon Kaen University, Khon Kaen, Thailand

E-mail address: apiporn@kku.ac.th (Apiporn T. Suwannatrai)

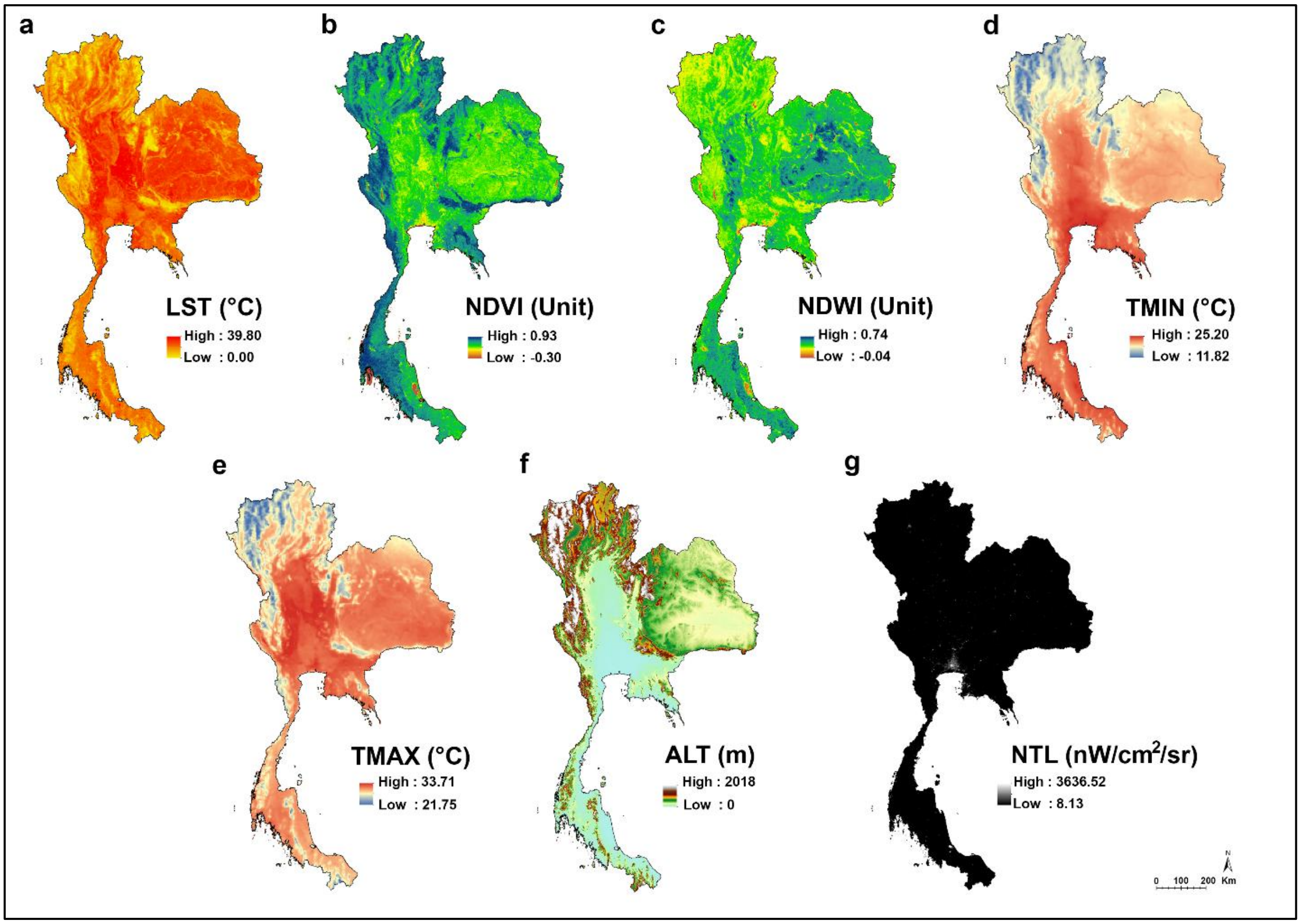

Fig. S1 Spatial distribution maps of environmental, climatic, and socio-economic factors across Thailand: (a) land surface temperature (LST), (b) normalized difference vegetation index (NDVI), (c) normalized difference water index (NDWI), (d) minimum temperature (TMIN), (e) maximum temperature (TMAX), (f) altitude (ALT), and (g) nighttime light intensity (NTL). Maps were generated by the authors using ArcGIS Pro version 3.3 (ESRI, Redlands, CA, USA; https://www.esri.com/en-us/arcgis/products/arcgis-pro/overview).

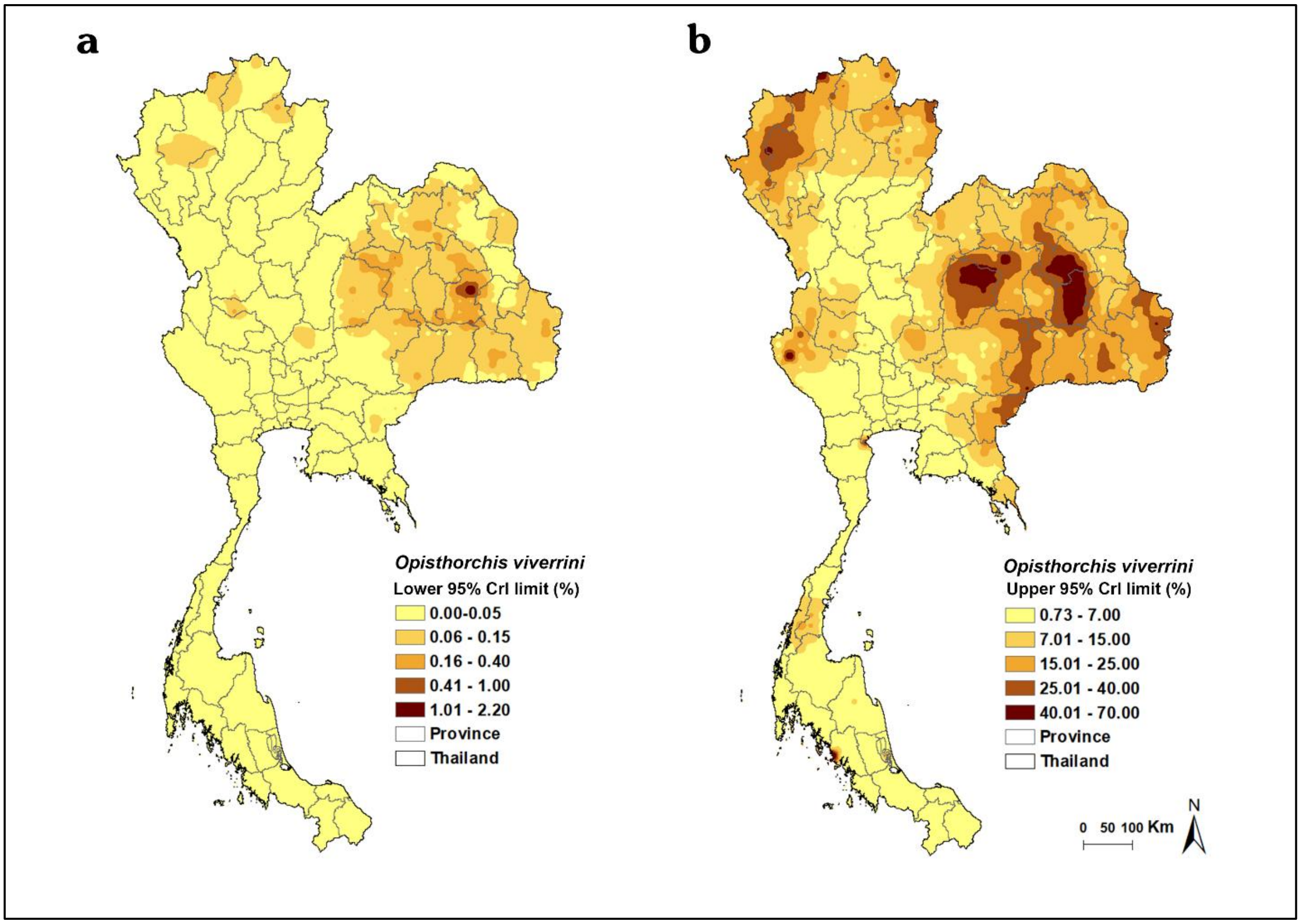

Fig. S2 (a) Lower and (b) upper 95% Bayesian credible interval (CrI) limits for predicted prevalence of *Opisthorchis viverrini* infection in Thailand. Maps were generated by the authors using ArcGIS Pro version 3.3 (ESRI, Redlands, CA, USA; https://www.esri.com/en-us/arcgis/products/arcgis-pro/overview).

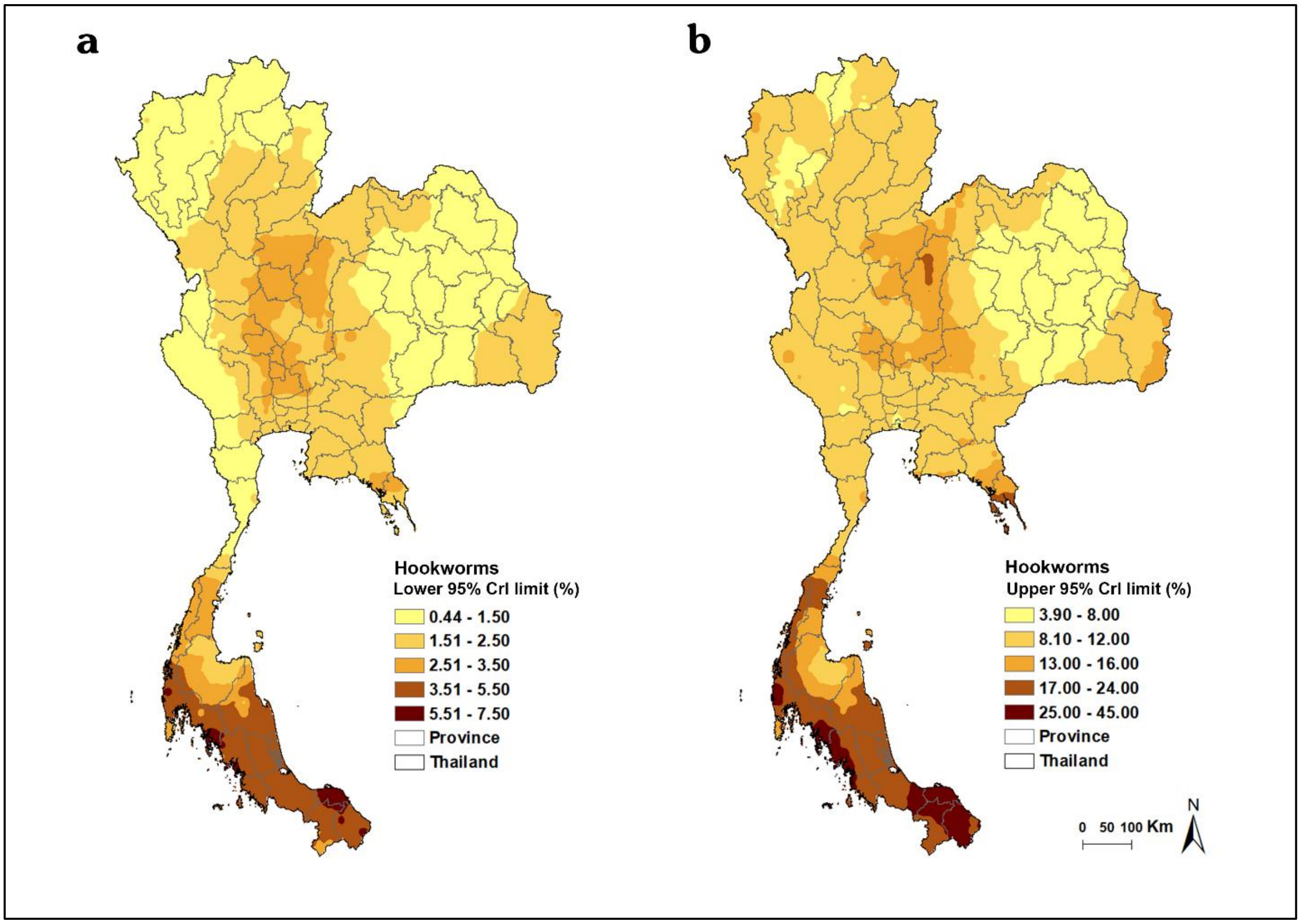

Fig. S3 (a) Lower and (b) upper 95% Bayesian credible interval (CrI) limits for predicted prevalence of hookworm infection in Thailand. Maps were generated by the authors using ArcGIS Pro version 3.3 (ESRI, Redlands, CA, USA; https://www.esri.com/en-us/arcgis/products/arcgis-pro/overview).

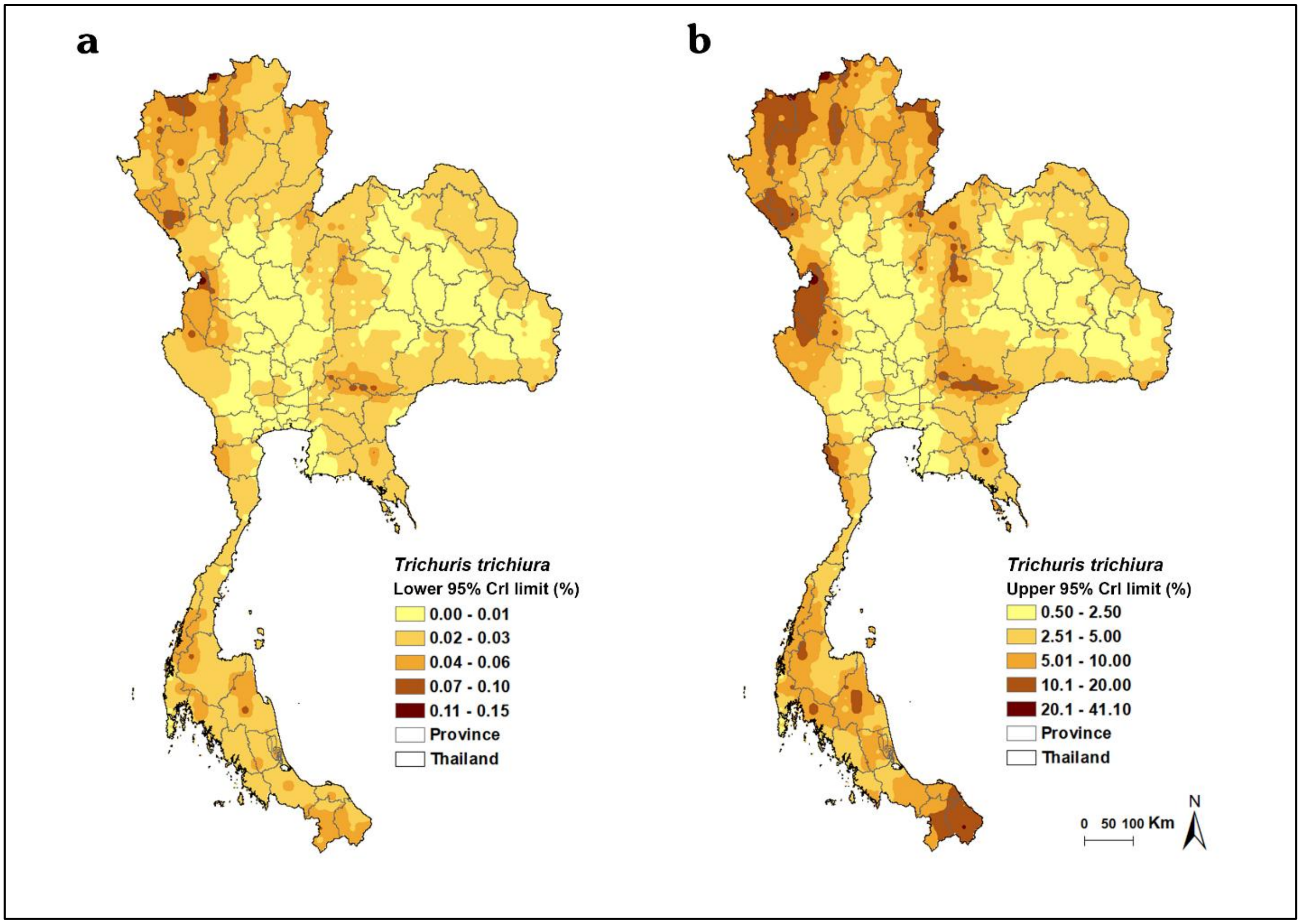

Fig. S4 (a) Lower and (b) upper 95% Bayesian credible interval (CrI) limits for predicted prevalence of *Trichuris trichiura* infection in Thailand. Maps were generated by the authors using ArcGIS Pro version 3.3 (ESRI, Redlands, CA, USA; https://www.esri.com/en-us/arcgis/products/arcgis-pro/overview).

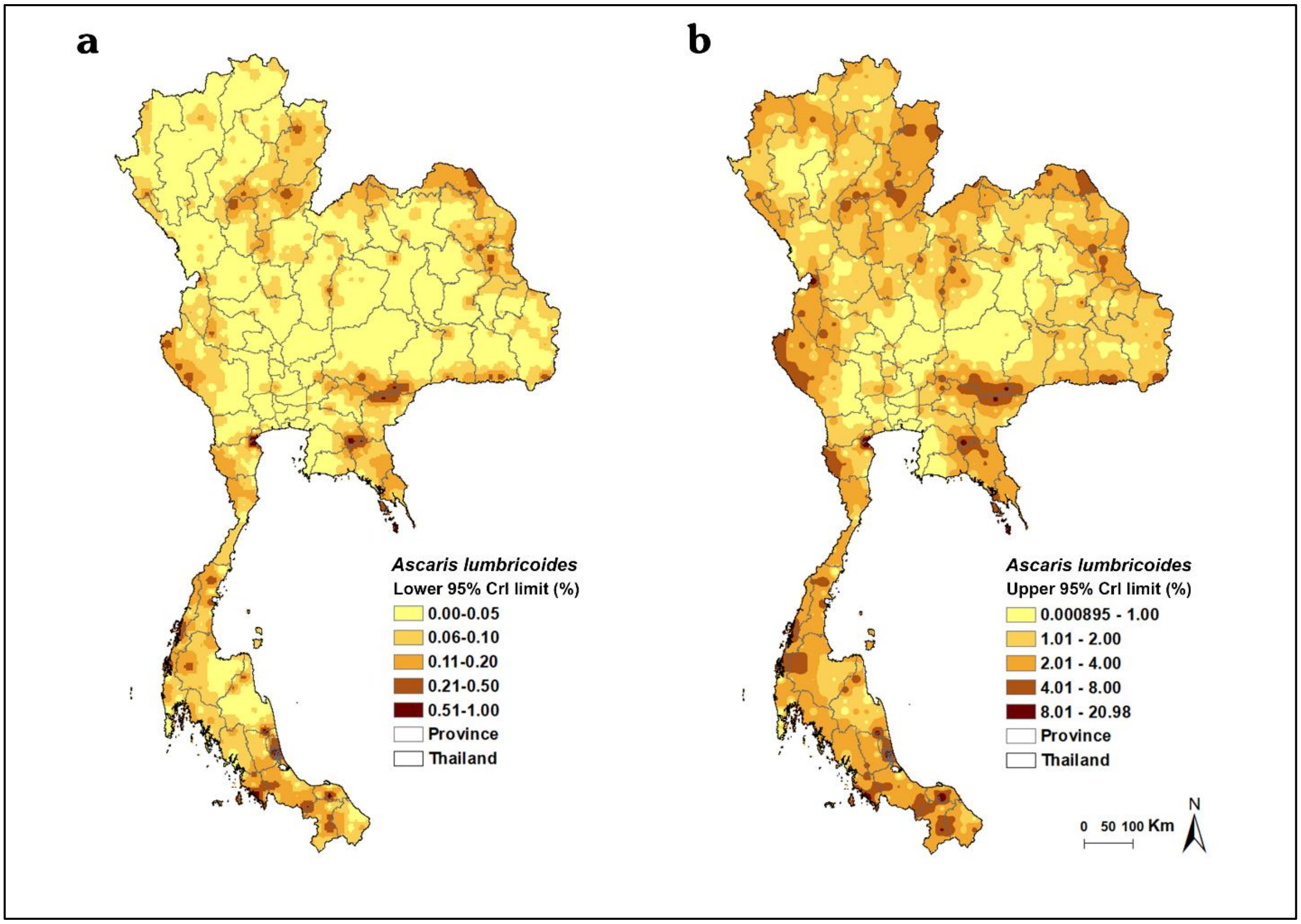

Fig. S5 (a) Lower and (b) upper 95% Bayesian credible interval (CrI) limits for predicted prevalence of *Ascaris lumbricoides* infection in Thailand. Maps were generated by the authors using ArcGIS Pro version 3.3 (ESRI, Redlands, CA, USA; https://www.esri.com/en-us/arcgis/products/arcgis-pro/overview).

Table S1 Comparison of unstructured (Model I) and structured (Model II) models for *Opisthorchis viverrini* infection based on Deviance Information Criterion (DIC) values.

Variables	**Model I: Unstructured model** OR (95% CrI)	**Model II: Structured model** OR (95% CrI)
Intercept (log odds scale)[a]	-5.03 (-5.42 -4.66)	-6.53 (-7.35 - 5.83)
Male (vs. female)	1.57 (1.27 1.94)	1.57 (1.24 -1.97)
25–59 years old (vs. age < 25 years old)	4.11 (2.85 6.10)	4.75 (3.22 - 7.21)
Age ≥ 60 years (vs. age < 25 years old)	3.87 (2.62 5.86)	4.69 (3.10 - 7.29)
NDVI	0.01 (0.00 0.04)	0.01 (0.00 - 0.25)
TMIN	0.78 (0.73 0.84)	0.67 (0.49 - 0.88)
NTL	0.99 (0.99 1.00)	1.00 (0.99 - 1.00)
DIC	1902.520	**1361.70***

[a]Coefficient, *best fit model, *NDVI* Normalized difference vegetation index *TMIN* Minimum temperature *NTL* Nighttime lights *DIC* Deviance information criterion

Table S2 Comparison of unstructured (Model I) and structured (Model II) models for hookworm infection based on Deviance Information Criterion (DIC) values

Variables	**Model I: Unstructured model** OR (95% CrI)	**Model II: Structured model** OR (95% CrI)
Intercept (log odds scale)[a]	-3.27 (-3.45 -3.09)	-3.30 (-3.69 - -2.83)
Male (vs. female)	1.63 (1.40 1.90)	1.65 (1.42 - 1.93)
25–59 years old (vs. age < 25 years old)	1.74 (1.43 2.12)	1.86 (1.52 - 2.28)
Age ≥ 60 years (vs. age < 25 years old)	1.37 (1.10 1.72)	1.41 (1.12 - 1.78)
LST	0.93 (0.89 0.96)	0.96 (0.91 - 1.02)
NDVI	2.52 (1.26 5.05)	0.95 (0.31 - 3.33)
TMIN	1.23 (1.17 1.30)	1.13 (0.95 - 1.30)
DIC	2929.520	**2773.19***

[a]Coefficient, *best fit model, LST: Land surface temperature, NDVI: Normalized difference vegetation index, TMIN: Minimum temperature, DIC: Deviance information criterion

Table S3 Comparison of unstructured (Model I) and structured (Model II) models for *Trichuris trichiura* infection based on Deviance Information Criterion (DIC) values

Variables	**Model I: Unstructured model** OR (95% CrI)	**Model II: Structured model** OR (95% CrI)
Intercept (log odds scale)[a]	-5.06 (-5.47 -4.67)	-6.09 (-6.76 - -5.49)
Male (vs. female)	1.02 (0.71 1.47)	0.97 (0.66 - 1.40)
25–59 years old (vs. age < 25 years old)	0.91 (0.59 1.41)	1.04 (0.67 - 1.64)
Age ≥ 60 years (vs. age < 25 years old)	1.07 (0.66 1.73)	1.16 (0.71 - 1.91)
LST	0.82 (0.74 0.90)	0.84 (0.70 - 1.09)
NDVI	0.21 (0.02 1.81)	0.12 (0.00 - 3.67)
TMAX	0.87 (0.75 1.03)	0.82 (0.55 - 1.12)
DIC	903.251	**775.10***

[a]Coefficient, *best fit model, LST: Land surface temperature, NDVI: Normalized difference vegetation index, TMAX: Maximum temperature, DIC: Deviance information criterion

Table S4 Comparison of unstructured (Model I) and structured (Model II) models for *Ascaris lumbricoides* infection based on Deviance Information Criterion (DIC) values

Variables	**Model I: Unstructured model** OR (95% CrI)	**Model II: Structured model** OR (95% CrI)
Intercept (log odds scale)[a]	-6.23 (-7.00 -5.54)	-11.44 (-16.83 - -8.07)
Male (vs. female)	1.29 (0.65 2.54)	1.18 (0.57 - 2.46)
25–59 years old (vs. age < 25 years old)	0.41 (0.18 0.91)	0.42 (0.18 - 0.98)
Age ≥ 60 years (vs. age < 25 years old)	0.61 (0.26 1.40)	0.76 (0.31 - 1.84)
LST	0.72 (0.61 0.83)	0.66 (0.39 - 0.98)
ALT	1.00 (0.99 1.00)	1.00 (0.99 - 1.00)
NTL	0.99 (0.98 1.00)	0.97 (0.93 - 0.99)
DIC	340.564	**199.22***

[a]Coefficient, *best fit model, LST: Land surface temperature, ALT: Altitude, NTL: Nighttime lights, DIC: Deviance information criterion
